# Color Image Quantization Algorithm Based on Self-Adaptive Differential Evolution

**DOI:** 10.1155/2013/231916

**Published:** 2013-07-15

**Authors:** Qinghua Su, Zhongbo Hu

**Affiliations:** ^1^School of Mathematics and Statistic, Hubei Engineering University, Xiaogan, Hubei 432000, China; ^2^School of Sciences, Wuhan University of Technology, Wuhan, Hubei 430070, China

## Abstract

Differential evolution algorithm (DE) is one of the novel stochastic optimization methods. It has a better performance in the problem of the color image quantization, but it is difficult to set the parameters of DE for users. This paper proposes a color image quantization algorithm based on self-adaptive DE. In the proposed algorithm, a self-adaptive mechanic is used to automatically adjust the parameters of DE during the evolution, and a mixed mechanic of DE and *K*-means is applied to strengthen the local search. The numerical experimental results, on a set of commonly used test images, show that the proposed algorithm is a practicable quantization method and is more competitive than *K*-means and particle swarm algorithm (PSO) for the color image quantization.

## 1. Introduction

Color image quantization, one of the common image processing techniques, is the process of reducing the number of colors presented in a color image with less distortion [1]. The main purpose of color quantization is reducing the use of storage media and accelerating image sending time [2]. Color image quantization consists of two essential phases. The first one is to design a colormap with a smaller number of colors (typically 8–256 colors [3]) than that of a color image. The second one is to map each pixel in the color image to one color in the colormap. Most of the color quantization methods focus on creating an optimal colormap. For being an NP-hard problem, it is not feasible to find the optimal colormap without a prohibitive amount of time [4]. To address this problem, researchers have applied several stochastic optimization methods, such as GA and PSO. In particular, the literature [5–8] has compared the color image quantization algorithm using PSO (PSO-CIQ) and several other well-known color image quantization methods. The experimental results show that PSO-CIQ has higher performance.

Differential evolution algorithm (DE) [9–11] is a population-based heuristic search approach. DE has been applied to the classification for gray images [12–14]. In the literature [12–14], DE and PSO show similar performance. However, due to simple operation, litter parameters, and fast convergence, DE is the better choice to use than PSO [12]. However, few researches have been done for using DE to solve the color image quantization. This paper applies DE to solve the color image quantization. However, the performance of DE is decided by two important parameters, the scaling factor *F* and the crossover rate CR. In practice, it is difficult to set the two parameters. For this difficulty, this paper proposes a color image quantization algorithm based on self-adaptive DE (SaDE-CIQ). In SaDE-CIQ, the self-adaptive mechanics in the literature [15, 16] are used to automatically adjust the parameters of DE during the evolution, and *K*-means is mixed into DE with a little probability for strengthening the local search. SaDE-CIQ starts with an initialized population, in which each individual represents a candidate colormap. A small number of candidate colormaps are adjusted by *K*-means. Then the adjusted candidate colormaps and the rest ones are repeatedly updated by DE operations, in which the parameters are automatically adjusted. The optimal solution is the optimal colormap, by which the quantized image is generated. By some commonly used color images, the performance of SaDE-CIQ in the color image quantization is compared with that of *K*-means and PSO.

This paper is organized as follows.  Section 2 introduces the classical DE briefly. In Section 3, a self-adaptive mechanic of DE parameters and a mixed mechanic of DE and *K*-means are introduced. In Section 4, SaDE-CIQ is proposed. In Section 5, numerical experiments are performed to compare the color image quantization qualities of SaDE-CIQ, *K*-means, and PSO.  Section 6 concludes this paper.

## 2. Classical Differential Evolution 

DE is a simple and powerful stochastic global optimization algorithm, and several DE variants or strategies have been presented. In this paper, we focus on the classical DE, which applies the simple arithmetic operations: mutation, crossover, and selection to evolve the population. Before the introduction of the classical DE, the following symbols used throughout this paper are defined:

- *g*(*x*): objective function or fitness function,
- *D*: the dimension of an optimization problem,
- *NP*: population size,
- *X* = {*x* ^1^, *x* ^2^,…, *x* ^*NP*^}: population,
- *x* ^*j*^ = (*x*_1_^*j*^, *x*_2_^*j*^,…, *x*_*D*_^*j*^): the *j*th individual in the population *X*, *j* = 1,2,…, *NP*,
- *F*: scaling factor,
- CR: crossover rate.

Consider the following optimization problem:

(1) min⁡g(x), x=(x1,x2,…,xD)∈∏i=1D[Li,Ui],i=1,2,…,D,

where *L*_*i*_ and *U*_*i*_ are the lower bound and upper bound of variable *x*_*i*_ and ∏_*i*=1_^*D*^[*L*_*i*_, *U*_*i*_] is the feasible domain of this problem.

An initial population *X* = {*x* ^1^, *x* ^2^,…, *x* ^*NP*^} including *NP* individuals is generated randomly, where each individual *x* ^*j*^ = (*x*_1_^*j*^, *x*_2_^*j*^,…, *x*_*D*_^*j*^), *j* = 1,2,…, *NP*.

The following mutation operation is performed to generate a donor vector for each individual *x* ^*j*^:

(2) uj=(u1j,u2j,…,uDj)=xr1+F·(xr2−xr3),j=1,2,…,NP,yij={Li,if  (uij<Li),Ui,if  (uij>Ui),i=1,2,…,D,j=1,2,…,NP,uij,otherwise,yj=(y1j,y2j,…,yDj), j=1,2,…,NP,

where *y* ^*j*^ is donor vector, *r*_1_, *r*_2_, and *r*_3_ are three uniformly different integers on [1, *NP*], and the scaling factor *F* is a parameter on [0,1].

Then, the following crossover operation is performed to obtain a trial vector for each individual *x* ^*j*^:

(3) $$\begin{array}{c} z^{j}=\left(z_{1}^{j},z_{2}^{j},\ldots ,z_{D}^{j}\right),\;j=1,2,\ldots ,NP,\\ z_{i}^{j}=\{\begin{array}{cc} y_{i}^{j}, & \text{if}\;\left(\text{ran}{\text{d}}_{i}\leq \text{CR}\;\text{or}\;i=\text{rnb}{\text{r}}_{j}\right)\\ x_{i}^{j}, & \text{if}\;\left(\text{ran}{\text{d}}_{i}>\text{CR}\;\text{and}\;i\neq \text{rnb}{\text{r}}_{j}\right),\\ & i=1,2,\ldots ,D, \end{array}\; \end{array}$$

where *z* ^*j*^ is trial vector, the crossover rate CR is a parameter on [0,1], rand_*i*_ is a uniformly random value on [0,1], and rnbr_*j*_ is a uniformly random integer on [1, *D*] for each different *j* to assure that at least one component of *z* ^*j*^ is taken from the donor vector.

Finally, according to the fitness values of the fitness function, the population is updated by the following selection operation:

(4) $$\begin{array}{c} {x^{j}}^{\prime }=\{\begin{array}{cc} x^{j}, & \text{if}\;\left(g\left(x^{j}\right)\leq g\left(z^{j}\right)\right)\\ z^{j}, & \text{if}\;\left(g\left(x^{j}\right)>g\left(z^{j}\right)\right),\\ & j=1,2,\ldots ,NP, \end{array}\;\; \end{array}$$

where *x* ^*j*^′ is the updated individual for the next generation population *X*′ = {*x* ^1^′, *x* ^2^′,…, *x* ^*NP*^′}.

As stated previously, for obtaining the best solution of the fitness function *g*(*x*), DE starts with a randomly generated initial population and repeatedly updates the population with the mutation, crossover, and selection operations until the stopping condition is satisfied.

## 3. A Self-Adaptive Mechanic and a Mixed Mechanic of DE

The scaling factor *F* and the crossover rate CR can influence the convergence and stability of DE. In practice, it is more difficult to set right *F* and CR for user. One of the effective methods to solve this difficult problem is to self-adaptively control the parameters in DE during evolution. In the following SaDE-CIQ, *F*  and CR are automatically updated for each individual in each generation by the self-adaptive mechanics of the literature [15, 16]:

(5) $$\begin{array}{c} F^{j}=\{\begin{array}{cc} 0.1+\text{rand}*0.9, & \text{if}\;\;\text{rand}<0.1,\\ F^{j}, & \text{otherwise}, \end{array} \end{array}$$

(6) $$\begin{array}{c} \text{C}{\text{R}}^{j}=\{\begin{array}{cc} \text{rand},\; & \text{if}\;\;\text{rand}<0.1,\\ \text{C}{\text{R}}^{j}, & \text{otherwise}. \end{array} \end{array}$$

Generally, self-adaptively adjusting parameters maybe have negative effect on the performance of DE. For improving the color quantization quality of self-adaptive DE, a mixed mechanic is used in the following SaDE-CIQ. *K*-means is a quickly cluster algorithm with better local search ability. In the mixed mechanic, a small number of individuals from a population are selected by a little probability *p*. Before being updated by DE, the selected individuals are adjusted by *K*-means quickly. The mixed operation can simplify the search space of DE and improve its convergence speed.

**Pseudocode 1:**
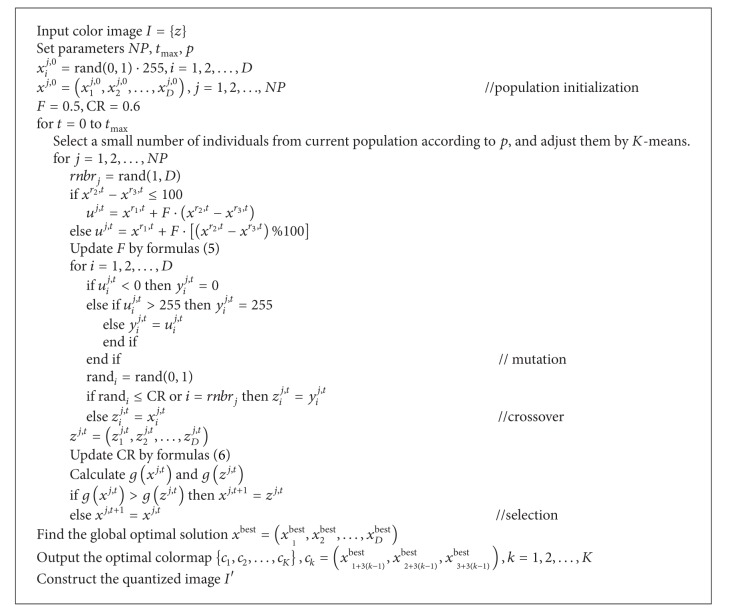
The pseudocode of the SaDE-CIQ.

## 4. Color Image Quantization Algorithm Based on Self-Adaptive DE

In RGB color space, each color pixel of a color image is a combination of red, green, and blue (RGB). For color images, the data space is [0,255]^3^. For a given color image *I*, the color number of the image *I* is set to be *N*, and the set of all colors belonging to *I* is set to be *S*.  *S*′, called a colormap, is a subset with  *K*  colors in [0,255]^3^, where *K* < *N*. The color image quantization is to design a colormap *S*′ and to create a map *f* : *S* → *S*′, by which each color pixel in *S* is replaced by one of the colors in *S*′. Thus, a new color image *I*′, called the quantized image of *I*, with the *K* colors in *S*′ is constructed. The objective to quantize the color image *I* is to minimize the color error between the color image *I* and its quantized image *I*′.

The color image quantization consists of two major phases:

- design a better colormap with a reduced number of colors (typically 8–256);
- create the mapping relationship between the color image and the colormap by which a quantized image is obtained.

In the process of color image quantization, it is most important to design an optimal colormap. To address this problem, researchers have applied some heuristic techniques for color image quantization. These techniques can be mainly categorized into preclustering approaches and postclustering approaches. Although being time consuming, the postclustering approaches are superior to the preclustering approaches in the quantization quality. Postclustering approaches perform clustering of the color space [17]. A postclustering algorithm starts with an initial colormap concluding *K* colors. Each color pixel of the image *I* is mapped to the color in the colormap with the minimal color distance from the color pixel. Thus, all the color pixels in image  *I*  are clustered into *K* clusters whose centers are separately the *K* colors in the colormap. Then the colormap and the *K*  clusters are iteratively modified to improve the optimum.

This section describes a new postclustering color image quantization approach using self-adaptive DE, called the color image quantization algorithm based on self-adaptive DE (SaDE-CIQ). A measure is given to quantify the quality of the resultant quantized image, after which the SaDE-CIQ is introduced.

### 4.1. Measure of Quality

The mean square error (MSE) is the most general measure of quality of a quantized image [4]. It represents the color error between image *I* and its quantized image *I*′. In the following SaDE-CIQ, the MSE is set to be the fitness function, which is defined as follows:

(7) $$\begin{array}{c} \text{MSE}=\frac{1}{N_{p}}\left\{\sum_{r=1}^{N_{p}}\left[\overset{K}{\underset{k=1}{\min}}d\left(p_{r},c_{k}\right)\right]\right\}, \end{array}$$

where the symbols used in (7), which will be used in the remaining parts of this paper, are explained as follows:

- *N*_*p*_: the number of image pixels,
- *K*: the color number in the colormap,
- *p*_*r*_ = (*p*_*r*1_, *p*_*r*2_, *p*_*r*3_): the *r*th pixel of the color image *I*, *r* = 1,2,…, *N*_*p*_,
- *c*_*k*_: the *k*th color triple in the colormap, *k* = 1,2,…, *K*.

### 4.2. Description of the SaDE-CIQ

In the SaDE-CIQ, the classical DE with the previously mentioned self-adaptive mechanic and mixed mechanic is used for the color image quantization. A population *X* = {*x* ^1^, *x* ^2^,…, *x* ^*NP*^} represents a set of candidate colormaps. Each individual represents a candidate colormap with *K*  color triples in the RGB color space [0,255]^3^. The*j*th individual is denoted by

(8) xj=(c  1j,c  2j,…,cKj) =(x1j,x2j,x3j,x4j,x5j,x6j,…,x3K−2j,x3K−1j,x3Kj),                 j=1,2,…,NP,

where *c*_*k*_^*j*^ = (*x*_1+3(*k*−1)_^*j*^, *x*_2+3(*k*−1)_^*j*^, *x*_3+3(*k*−1)_^*j*^), *k* = 1,2,…, *K*. Thus, the dimension of each individual is *D* = 3 × *K*, and the feasible domain is [0,255]^3×*K*^. The quality of each individual is measured by the MSE in (7):

(9) g(xj)=MSE(xj)=1Np{∑r=1Np[min⁡k=1Kd(pr,ckj)]}=1Np{∑r=1Np[min⁡k=1K∑q=13(prq−xq+3(k−1)j)2]},j=1,2,…,NP.

The stopping condition of the algorithm is to reach a specified maximal number of iterations *t*_max⁡_.

In the first phase of the SaDE-CIQ, an optimal colormap is designed. A set of *NP* candidate colormaps are initialized. Each colormap consists of *K* randomly selected color triples in the color space [0,255]^3^. *K*-means is applied to adjust a small number of colormaps randomly selected from all the candidate colormaps by a little probability *p*. Then the adjusted colormaps and the rest ones are repeatedly updated by mutation and crossover operations, where *F* and CR with the initial values 0.5 and 0.6 are updated by formulas (5) and (6). The *K*-means and DE operations are performed until the stopping condition is satisfied. The last optimal solution is the optimal colormap. In the second phase of the SaDE-CIQ, the mapping relationship is created according to the minimal color distance principle. By replacing each pixel in the color image *I* with its corresponding color in the optimal colormap, *I* is to be reconstructed to obtain the quantized image *I*′.

See Pseudocode 1 of the SaDE-CIQ.

## 5. Numerical Experiments

In this section, the SaDE-CIQ is tested on a set of four commonly used test images in the quantization literature. In addition, the performance of the SaDE-CIQ is compared with that of *K*-means and the color image quantization algorithm using PSO (PSO-CIQ) presented in literature the [5].

### 5.1. Images and Parameters Set

**Figure 1:**
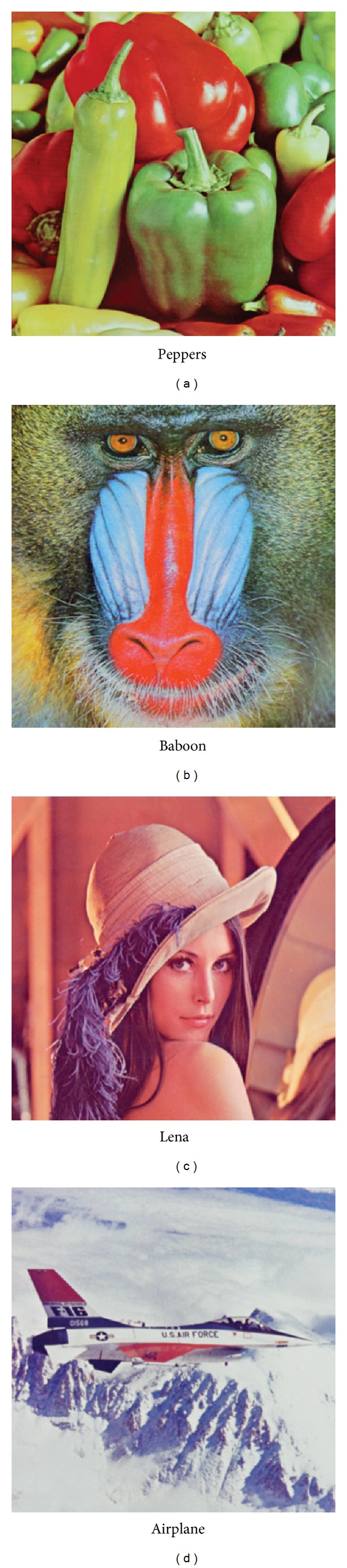
Test images.

The set of test images include Lena, Peppers, Baboon, and Airplane, which have the same size 512 × 512 pixels. They are shown in Figure 1.

The parameters in the SaDE-CIQ are set as the population size  *NP* = 100, the maximal number of iterations *t*_max⁡_ = 200, and the mixed probability *p* = 0.05.

The PSO-CIQ has more parameters than the SaDE-CIQ. They are set as the swarm size *NP* = 100, the inertia weight *ω* = 0.72, the acceleration constants *c*_1_ = *c*_2_ = 1.49, the maximum velocity *V*_max⁡_ = 0.4, and the maximal number of iterations *t*_max⁡_ = 200. These parameters except for the last one are as same as those in the literature [5]. 

### 5.2. Experimental Results

**Figure 2:**
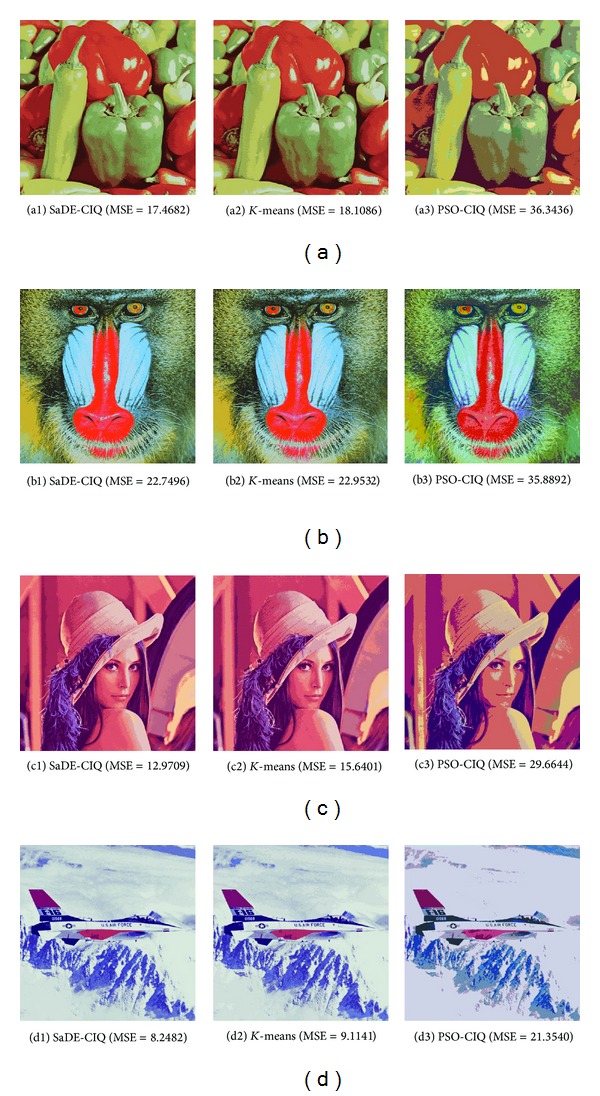
The quantized images obtained by SaDE-CIQ, *K*-means, and PSO-CIQ.

**Figure 3:**
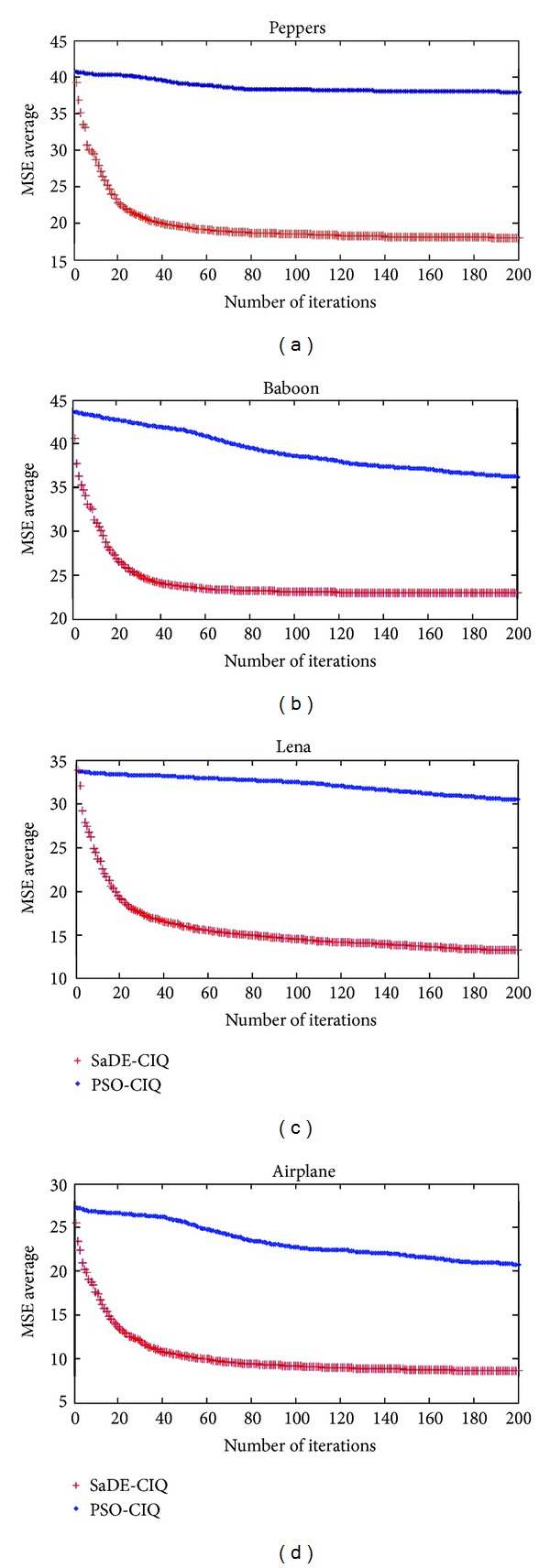
The average MSE variations with the number of iterations of SaDE-CIQ and PSO-CIQ.

For each algorithm, the test images are quantized into 16 colors. The colors quantized images with the smallest MSEs over 10 simulations are shown in Figure 2. The smallest MSEs and the largest MSEs over 10 simulations are listed in Table 1, and for SaDE-CIQ and PSO-CIQ, the MSE variations with the number of iterations are exhibited in Figure 3.

### 5.3. Analysis of Experimental Results

As shown in Figure 2, the SaDE-CIQ outperforms *K*-means and PSO-CIQ in the visual quality of the quantized images for all test images. The quantized images a-1, b-1, c-1, and d-1 have richer layers and more details than the other quantized images.

As illustrated in Table 1, the SaDE-CIQ generates a smaller MSEs than *K*-means and PSO-CIQ for each test image.

Shown in Figure 3, the SaDE-CIQ has a smaller average MSE than the PSO-CIQ at each same number of iterations. Moreover, the average MSE resulting from the SaDE-CIQ decreases more quickly than that resulting from the PSO-CIQ with the increasing number of iterations.

The above experimental results can be summarized as follows:

- the SaDE-CIQ is an effective color image quantization method;
- the SaDE-CIQ has better quantization quality than *K*-means and PSO-CIQ;
- the SaDE-CIQ converges more quickly than the PSO-CIQ.

## 6. Conclusions

This paper presents a color image quantization algorithm based on self-adaptive DE (SaDE-CIQ). Numerical experiments are implemented to investigate the performance of the SaDE-CIQ and to compare it against *K*-means and PSO-CIQ presented in the literature [5]. For a set of commonly used test images, the experimental results demonstrate the feasibility of the SaDE-CIQ and its superiority to *K*-means and PSO-CIQ in the quantization quality. In addition, the SaDE-CIQ has simpler operation, litter parameters, and faster convergence than the PSO-CIQ.

## Figures and Tables

**Table 1:** The MSEs resulting from SaDE-CIQ and PSO-CIQ.

Alg.	Peppers		Baboon		Lena		Airplane	
	min	max	min	max	min	max	min	max
SaDE-CIQ	17.4682	18.7266	22.7496	23.3382	12.9709	13.8055	8.2482	8.9740
*K*-means	18.1086	21.2676	22.9532	24.9563	15.6401	19.1314	9.1141	10.4430
PSO-CIQ	36.3436	40.9532	35.8892	41.9940	29.6644	34.5867	21.3540	24.3200
